# Efficacy of Subdermal Poly‐d,l‐Lactic Acid Injections for the Treatment of Melasma

**DOI:** 10.1111/jocd.16650

**Published:** 2024-10-27

**Authors:** Jovian Wan, Kyu‐Ho Yi

**Affiliations:** ^1^ Medical Research Inc. Wonju Korea; ^2^ Division in Anatomy and Developmental Biology, Department of Oral Biology, Human Identification Research Institute, BK21 FOUR Project Yonsei University College of Dentistry Seoul Korea; ^3^ Maylin Clinic (Apgujeong) Seoul Korea

**Keywords:** collagen stimulation, dermal remodeling, melasma, PDLLA, poly‐d,l lactic acid, skin pigmentation

## Abstract

**Background:**

Melasma is a chronic, recurrent skin disorder with limited long‐term treatment success using conventional therapies like hydroquinone and laser treatments, which primarily target epidermal components while leaving dermal aspects untreated.

**Objective:**

To evaluate the efficacy and safety of poly‐d,l lactic acid (PDLLA) subdermal injections for treating moderate melasma.

**Methods:**

Three female patients (age range: 45–59 years) with Fitzpatrick skin types III and IV received three PDLLA injection sessions at 3‐week intervals. Treatment outcomes were assessed using the Melasma Area and Severity Index (MASI) and patient satisfaction scores at 12‐week follow‐up.

**Results:**

All patients showed significant MASI score improvements (reduction range: 3.60–6.30 points). Patient satisfaction ratings ranged from 3 to 4 out of 4. Temporary side effects included mild edema and bruising, resolving within 72 h.

**Conclusions:**

PDLLA subdermal injections showed promising results in melasma treatment, potentially due to its biostimulatory effects on collagen production and dermal remodeling. Further research, including histopathological analysis, is needed to confirm long‐term efficacy and safety, and understand underlying mechanisms.

## INTRODUCTION

Melasma is a chronic condition that poses significant management challenges due to its recurrent nature and the limited effectiveness of standard treatments, which often result in inconsistent outcomes and high relapse rates. Conventional options, including hydroquinone and laser therapy, generally target superficial layers of the skin, leaving deeper dermal components unaddressed [1, 2]. In this case series, we explore the use of poly‐d,l‐lactic acid (PDLLA) administered via subdermal needle injection as a potential therapeutic intervention for moderate melasma, targeting both superficial and deep dermal components.

## CASE PRESENTATION

### Case 1

A 59‐year‐old woman with Fitzpatrick skin type III, presented with moderate melasma affecting her cheeks. Her baseline melasma area and severity index (MASI) scores were 15.60 and 17.20, as assessed by two independent, blinded dermatologists. The patient had previously undergone multiple unsuccessful treatments, including laser therapy and chemical peels. She received three sessions of subdermal PDLLA injections (Juvelook, VAIM Global, South Korea) at 3‐week intervals, targeting the areas of hyperpigmentation.

**FIGURE 1:**
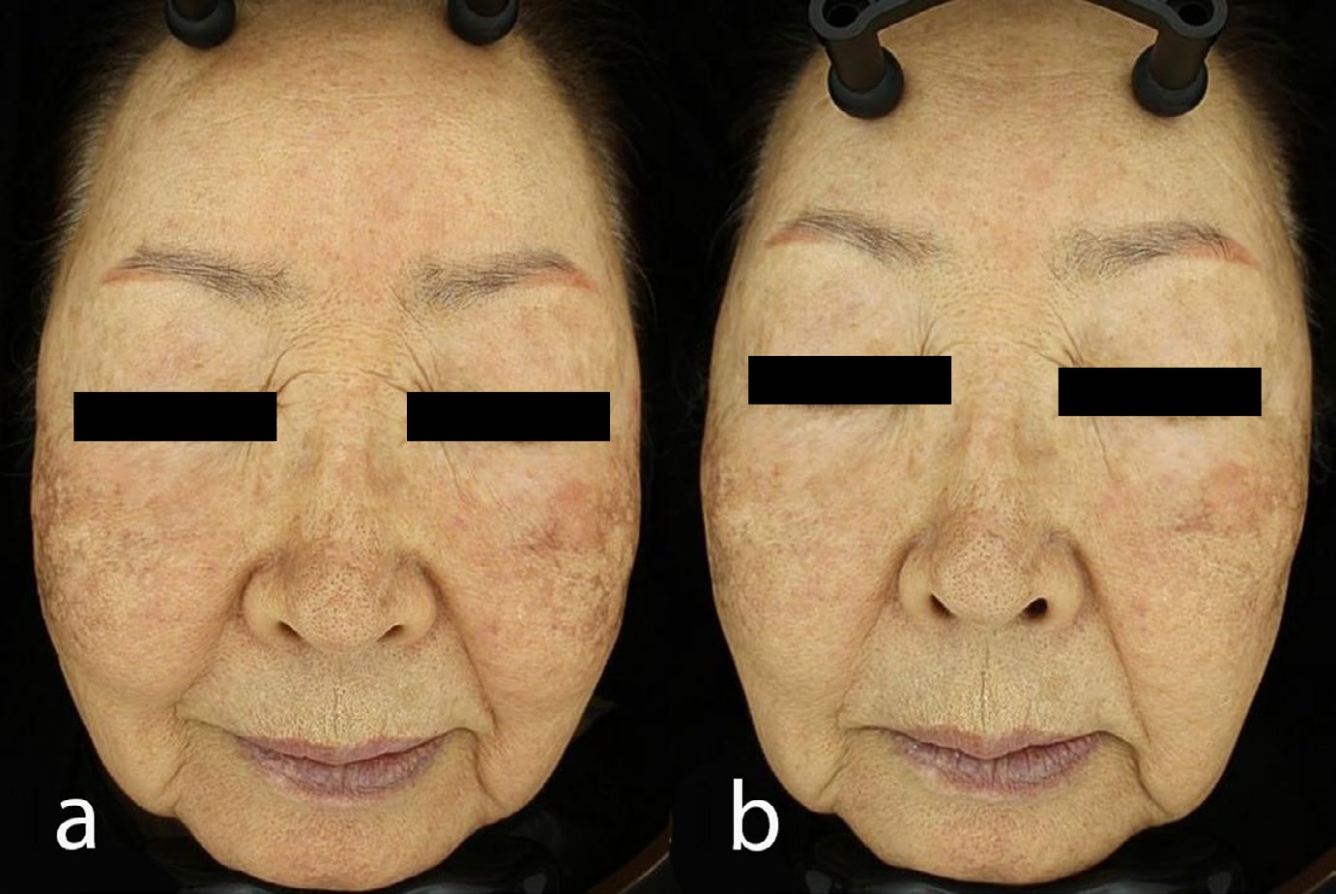
A 59‐year‐old woman with moderate melasma primarily affecting her cheeks. She had previously undergone multiple treatments, including laser therapy and chemical peels, without significant improvement. Figure (a) shows the patient's skin condition at baseline, with MASI scores of 15.60 (Dermatologist 1) and 17.20 (Dermatologist 2). Figure (b) illustrates the notable improvement at the 12‐week follow‐up, where the MASI scores decreased to 10.80 (Dermatologist 1) and 13.40 (Dermatologist 2) after three sessions of poly‐d, l‐lactic acid injections. The patient reported a substantial reduction in hyperpigmentation, a more uniform skin texture, and high satisfaction with the results. Mild edema observed post‐treatment resolved spontaneously without complications.

At the 12‐week follow‐up, the patient showed marked improvement in melasma severity, with MASI scores reduced to 10.80 and 13.40, indicating a substantial decrease in hyperpigmentation (Figure 1). The patient rated her satisfaction as 4 out of 4, reflecting a high level of contentment with the treatment results. Minor adverse effects, including mild swelling and bruising, were noted post‐treatment but resolved within 48–72 h without complications (Table 1).

**TABLE 1:** Summary of patient outcomes.

Patient	Age	Baseline MASI score (Dermatologist 1)	Baseline MASI score (Dermatologist 2)	MASI Score at 12 weeks (Dermatologist 1)	MASI Score at 12 weeks (Dermatologist 2)	Adverse Events	Patient satisfaction score (0–4)
1	59	15.60	17.20	10.80	13.40	Mild oedema, resolved spontaneously, no other complications	4
2	45	18.30	19.10	12.40	14.0	Mild swelling and bruising, resolved in 48 hours	4
3	50	16.20	17.50	11.90	13.1	Mild oedema, resolved in 72 hours	3

### Case 2

A 45‐year‐old woman with Fitzpatrick skin type III and a five‐year history of recurrent melasma presented with moderate to severe melasma on the forehead and cheeks. Her initial MASI scores were 18.30 and 19.10. After unsuccessful treatments with hydroquinone and chemical peels, she underwent three sessions of subdermal PDLLA injections at three‐week intervals. By the 12‐week follow‐up, her MASI scores had improved to 12.40 and 14.00. The patient expressed high satisfaction, noting significant lightening of hyperpigmented areas. Minimal swelling and bruising were reported post‐injection but resolved within 48 hours.

### Case 3

A 50‐year‐old woman with Fitzpatrick skin type IV, previously treated with laser therapy and oral tranexamic acid for persistent melasma, presented with moderate melasma on her cheeks and upper lip. Her baseline MASI scores were 16.20 and 17.50. Following three sessions of PDLLA injections spaced three weeks apart, her MASI scores reduced to 11.90 and 13.10 at the 12‐week follow‐up. The patient reported notable improvement in the uniformity of skin tone and expressed satisfaction with the treatment, rating it 3 out of 4. Mild oedema was observed post‐treatment, resolving within 72 hours.

## DISCUSSION

PDLLA is a biodegradable polymer with collagen‐stimulating properties, traditionally used for volumising and contouring. Its biostimulatory effects suggest potential benefits for addressing the deeper dermal layers involved in melasma. By targeting the dermal layer, PDLLA could remodel the dermal microenvironment and reduce dermal hypermelanosis. This is important as melasma is thought to have a dermal component, with increased vascularization, inflammation, and melanophages contributing to its persistence. Conventional treatments that target the epidermis often fail to address these underlying factors, which may explain the high recurrence rates associated with superficial treatments [3].

The observed improvement may be attributed to PDLLA's mechanism of action, which involves inducing a controlled inflammatory response, stimulating fibroblasts, and promoting neocollagenesis [4]. These processes lead to the remodeling of the dermal matrix and enhanced skin resilience, aligning with previous findings that demonstrate PDLLA's efficacy in improving skin structure and texture through collagen synthesis. However, without histopathological evidence, it is unclear if the improvement in melasma was directly related to dermal remodeling. Further research including histological assessments is needed to confirm this hypothesis.

Despite the promising outcome, this case report has limitations, including a small sample size and a short follow‐up period, which restrict the generalizability of the results. Additionally, the absence of histological analysis prevents a deeper understanding of the precise mechanisms at play. Future studies involving larger, more diverse populations and longer follow‐up periods, combined with histological assessments, are essential to confirm the long‐term efficacy and safety of PDLLA for melasma treatment.

In conclusion, PDLLA injections offer a promising therapeutic option for patients with melasma by addressing both superficial and deeper components of the condition. While the precise mechanisms need further exploration, this case contributes to the growing body of evidence supporting biostimulatory agents like PDLLA as an innovative approach to treating pigmentation disorders.

## Author Contributions

All authors have reviewed and approved the article for submission. Conceptualization: Kyu‐Ho Yi. Study design: Jovian Wan. Acquisition of data: Jovian Wan. Writing – Original draft preparation: Jovian Wan, Kyu‐Ho Yi. Writing: Jovian Wan, Kyu‐Ho Yi. Visualization: Kyu‐Ho Yi, Jovian Wan. Supervision: Kyu‐Ho Yi.

## Conflicts of Interest

I acknowledge that I have considered the conflict of interest statement included in the “Author Guidelines.” I hereby certify that, to the best of my knowledge, that no aspect of my current personal or professional situation might reasonably be expected to significantly affect my views on the subject I am presenting.

## Data Availability

The data that support the findings of this study are available from the corresponding author upon reasonable request.

## References

[1] A. C. Handel , L. D. Miot , and H. A. Miot , “Melasma: A Clinical and Epidemiological Review,” Anais Brasileiros de Dermatologia 89, no. 5 (2014): 771–782. 25184917 10.1590/abd1806-4841.20143063 PMC4155956

[2] M. X. Wu , R. Antony , and H. N. Mayrovitz , “Melasma: A Condition of Asian Skin,” Cureus 13, no. 4 (2021): e14398. 33987053 10.7759/cureus.14398 PMC8110291

[3] T. K. Noh , S. J. Choi , B. Y. Chung , et al., “Inflammatory Features of Melasma Lesions in Asian Skin,” Journal of Dermatology 41, no. 9 (2014): 788–794. 25132344 10.1111/1346-8138.12573

[4] S. Oh , S. B. Seo , G. Kim , S. Batsukh , K. H. Son , and K. Byun , “Poly‐d,l‐Lactic Acid Stimulates Angiogenesis and Collagen Synthesis in Aged Animal Skin,” International Journal of Molecular Sciences 24, no. 9 (2023): 7986. 37175693 10.3390/ijms24097986 PMC10178436

